# PRSS22 promotes the immune evasion of gastric cancer via inhibiting ANXA1-mediated degradation of PD-1

**DOI:** 10.29219/fnr.v69.13155

**Published:** 2025-12-18

**Authors:** Jifan Wang, Jing Zhou, Xiaoping Chen

**Affiliations:** Department of Cancer Diagnosis and Treatment Center, Affiliated Hospital of Jiangnan University, Wuxi 214122, China

**Keywords:** gastric cancer, PRSS22, immune evasion, ANXA1, PD-1

## Abstract

**Purpose:**

Gastric cancer (GC) ranks as the third most prevalent cause of mortality among malignant tumors globally. This study endeavors to uncover the protective function and underlying mechanism of PRSS22 on the immune evasion of GC.

**Methods:**

A total of 24 GC patients and 7 normal volunteer were recruited. The gene chip data GSE291766 in GC was downloaded to analyze the differentially expressed genes (DEGs). DAVID database was used to perform the gene ontology (GO) and Kyoto Encyclopedia of Genes and Genomes (KEGG) enrichment analysis. TIMER database was used to quantify PRSS22 in different cancers as well as the correlation between the PRSS22 expression and the abundance of immune infiltration by gene module. GEPIA2 was used to analyze the prognostic significance of PRSS22 in GC patients. GC cells (NCI-N87, MKN-45, MKN-28, and AGS) were used to compare the PRSS22 expression with GES-1. Jurkat cells transfected with negative, PRSS22, si-NC, or si-PRSS22 were cultivated with AGS to establish the in vitro coculture system. All mice were inoculated with AGS and injected with negative or sh-PRSS22 lentivirus. qPCR was used to analyze the PRSS22, TP53, Cox2, MYC, TNF-α, CD28, GLUT1, Granzyme B, TCF-1, LAG-3, NR4A, and TIM-3 mRNA levels. Western blotting was used to measure the PRSS22, ANXA1, and PD-1 proteins levels. The commercial kits were used to determine the caspase-3/7/9, LDH, TGF-β, TNF-α, IFNγ, and IL-10 levels. Cell viability, migration, and proliferation were determined using CCK-8, PI fluorescent stain, transwell, and EdU assays. TNFα/CD8 and CD4^+^CD25^+^FoxP3^+^ levels were measured by flow analysis. In vivo imaging and immunohistochemical analysis were used to detect the ANXA1 expression. Immunofluorescence analysis was used to determine the PRSS22 and ANXA1 expressions. IP assay was used to analyze the interaction of PRSS22 protein with ANXA1 protein along with PD-1 ubiquitination.

**Results:**

PRSS22 was highly expressed in GC patients. The overall survival in GC patients (high-PRSS22) was lower than GC patients (low-PRSS22). PRSS22 mRNA and protein expressions were significantly upregulated in AGS cells. In the GC mouse model, PRSS22 downregulation decreased tumor volume and weight. PRSS22 was manifested in T cells of GC patients. In coculture with AGS and Jurkat cell, PRSS22 could significantly promote cell proliferation, migration, and EdU positivity but reduced LDH activity and PI levels. We discovered that the PRSS22 promoted the immune evasion in T cells of GC. ANXA1 and PD-1 were DEGS in sh-PRSS22 GC samples, and ANXA1/PD-1 pathway might be important for the functions of PRSS22 on immune evasion in the GC model. PRSS22 knockout upregulated ANXA1 but downmodulated PD-1 in *in vitro* and *in vivo* experiments. PRSS22 WT protein could interact with the ANXA1 WT protein, and ANXA1 upregulation could promote the ubiquitination of PD-1 protein. In coculture with AGS and Jurkat cell, si-PRSS22 treatment could reduce the tumor cell growth, migration, Edu positivity, and the immune evasion ability, which would be reversed by suppressing ANXA1.

**Conclusion:**

PRSS22 suppressed ANXA1-mediated degradation of PD-1 in T cell to promote the immune evasion of GC. Targeting PRSS22 is thus a potentially effective therapeutic strategy for GC.

## Popular scientific summary

- Gastric cancer is the third leading cause of cancer death worldwide, and its treatment faces enormous challenges.
- A recent study has discovered that a protein called PRSS22 plays a crucial role in the immune evasion of gastric cancer, providing new insights into its treatment.

According to the GLOBOCAN 2022 report, gastric cancer (GC) ranked 5th globally in both incidence and mortality (1). There were 969,000 new cases and 660,000 deaths, accounting for 4.9% of all cancer incidences and 6.8% of all cancer deaths. In China, new GC cases accounted for 37.0% of the global total, and deaths accounted for 39.4%, indicating that the overall burden remains relatively severe (2). Relevant studies have shown that the number of new cases and deaths of GC in China increased from 1990 to 2019. However, during the same period, the Age-Standardized Incidence Rate by China Standard Population (ASIRC) and the Age-Standardized Mortality Rate by Chinese Standard Population (ASMRC) decreased (3). Additionally, the incidence and mortality rates of GC in men were higher than women (3). Data from national cancer registration areas showed that in 2022, there were 358,700 new GC cases and 260,400 deaths, ranking 4th in both incidence and mortality spectra (4). The ASIRC was 13.79 per 100,000, and the ASMRC was 9.49 per 100,000. The ASIRC and ASMRC in men were 2.29 and 2.54 times than in women, respectively. In rural areas, the ASIRC and ASMRC were 1.20 and 1.33 times than in urban areas, respectively (5). It indicates that the incidence and mortality of GC are higher in men than in women and higher in rural areas than in urban areas (6).

Although the body’s immune system has the functions of immune surveillance, defense, and elimination, tumors can still cause severe damage to the human body (7). Studies have confirmed that tumors are not only the result of oncogene and tumor suppressor gene mutations and abnormal cell over-proliferation but also a systemic disease (7, 8). The body’s immune system and its microenvironment play an undeniable role in the occurrence and progression of tumors. Tumor cells evade recognition and attack by the body’s immune system through various mechanisms, including molecular mechanisms of tumor antigen recognition, tumor resistance to apoptosis, and tumor suppressive molecular mechanisms (9). Currently, tumor patients mainly receive multidisciplinary treatments such as surgery, radiotherapy, and chemotherapy. However, anticancer treatments for the advanced or recurrent tumors have certain limitations (10). There is an urgent need to develop new treatment methods to improve patients’ quality of life, and tumor immunotherapy has great potential in clinical tumor treatment (11).

Annexins (ANXA) are a class of calcium-dependent phospholipid-binding proteins that are involved in physiological and pathological processes such as anti-inflammation, regulation of cell proliferation, cell cycle regulation, DNA damage repair, growth factor signal transduction, formation of malignant tumors, and chemotherapy sensitivity (12). ANXA1 and ANXA2 are important members of the annexin family (13). Studies have found that the expression of ANXA1 is downregulated in ovarian cancer, and the overexpression of ANXA1 is an independent predictor of prolonged overall survival (OS) in ovarian cancer patients (12, 14). It is also downregulated in the human bladder cancer adriamycin-resistant cell line pumc-91/ADM, which is related to drug resistance in bladder cancer (15). The increased ANXA2 mRNA in stage III serous ovarian cancer is associated with the reduced survival. The ANXA2 in SGC7901/cisplatin cells is higher than parental SGC7901 cells. However, after knocking out ANXA2, the drug sensitivity of SGC7901/cisplatin cells to adriamycin, 5-fluorouracil (5-FU), and cisplatin is increased (16).

PRSS22 is known as tryptase ε and a member of the serine protease S1 family 22 (17). It is expressed in various malignant tumors such as glioma, pancreatic cancer, prostate cancer, ovarian cancer, breast cancer, and non-small cell lung cancer (18). In various tumor cells such as ovarian cancer, lung cancer, and rectal cancer, the PRSS22 expression is higher than the corresponding normal cells, suggesting that its abnormal expression may be related to the occurrence and development of tumors (19, 20). However, the research focusing directly on the mechanism or clinical significance of PRSS22 in GC has not been found yet. Therefore, this study attempts to reveal the protective role and mechanism of PRSS22 on the immune evasion of GC.

## Materials and methods

### Clinical samples

Patients with GC (*n* = 24) and normal volunteer (*n* = 7) were recruited from our hospital. This study was approved by the Ethics Committee of our hospital. Serum or tissue samples were collected and immediately stored at –80°C for subsequent analysis.

### Bioinformatics analysis

The gene chip data GSE291766 in GC was downloaded from National Center of Biotechnology Information (NCBI). R language analysis was used to harvest the differentially expressed genes (DEGs). The gene ontology (GO) and Kyoto Encyclopedia of Genes and Genomes (KEGG) enrichment analysis of DEGs were achieved through the DAVID online database. By the TIMER database (https://cistrome.shinyapps.io/timer/), we analyzed the PRSS22 expression in different cancer types as well as the correlation between the expression of PRSS22 and the abundance of immune infiltration, including T cells and macrophages by gene module. Prognostic significance of PRSS22 in GC patients was analyzed by GEPIA2 software (http://gepia2.cancerpku.cn/).

### Cell culture and transfection

GES-1, NCI-N87, MKN-45, MKN-28, and AGS cells were cultured in RPMI 1640 (Gibco) supplemented with 10% FBS (Gibco). Jurkat cell, an immortalized line of human T lymphocyte cell, was transfected with negative, PRSS22, si-NC, or si-PRSS22 using Lipofectamine 3,000 (Invitrogen, CA). Jurkat cells were stimulated with anti-CD3/CD28 antibodies (1 μg/mL each) + IL-2 (50 U/mL) for 48 h. AGS cells were seeded into 24-well plates (2 × 10 cells/well) and cultured for 24 h until 70% confluence. The activated Jurkat cells were added to AGS wells (CaSki:Jurkat = 1:2). AGS cells were seeded in the lower chamber, and activated Jurkat cells were seeded in the upper chamber.

### Vivo model establishment

The animal studies were authorized by the Animal Ethic Review Committees of our hospital. All animal experiments were strictly implemented in compliance with the NIH Guide for the Care and Use of Laboratory Animals. All mice were inoculated with AGS cells (1 × 10^7^ cells). Then, negative or sh-PRSS22 lentivirus (10^9^ PFU/mL, 200 mL) was injected into the mice via the tail vein.

### qPCR

Total RNAs were isolated with RNA isolator total RNA extraction reagent (Takara), and cDNA was synthesized using the PrimeScipt RT Master Mix (Takara). qPCR was performed with the ABI Prism 7,500 sequence detection. Relative levels of the sample mRNA expression were calculated.

### Histological, immunohistochemical, and immunofluorescence analyses and electron microscopy

For immunohistochemical and immunofluorescence analyses, mouse tissue samples were fixed in 4% paraformaldehyde and stained with hematoxylin and eosin (HE) as described in previous studies (21). Tissue samples were observed under a fluorescence microscope (Zeiss Axio Observer A1, Germany) and a transmission electron microscope (80 kV) (Hitachi H7650, Tokyo, Japan), as described in a previous study (22).

### ELISA, CCK8, EdU, and migration assays

TNF-α (PT512), IFNγ (PI508), IL-10 (PI522), and TGF-β (PT878) were performed as described in a previous study (23). Cell viability was determined using CCK-8 assay (C0037, Beyotime), as described in a previous study (22). Absorbance was measured on the Microplate Reader (Bio Tek, Winooski). EdU kit (C0075S, Beyotime) or LDH activity (C0016, Beyotime), Caspase3 (G01513), 4-HNE (H268-1-2), CAT (A007-1-1), Caspase7 (H080), and caspase9 (G01811) were quantified using the Commercial reagent kit (Nanjing Jiancheng Bioengineering Research Institute), and absorbance was measured at 450 nm using a fluorescent reader (Synergy H1 Microplate Reader, Bio Tek, Winooski). The migration ability of cells was detected through Transwell inserts (Corning, United States). 5×10^4^ cells/well were inoculated in the upper chamber with 200 μL of medium (free of FBS) and in the lower chamber with 600 μL of complete medium (10% FBS). After 48-h incubation, the migrated cells were fixed by adding 4% paraformaldehyde, stained by employing crystal violet, and captured under an inverted microscope (Olympus, Japan).

### Western blotting analysis and immunofluorescence

Western blotting analysis and immunofluorescence were executed as per the literature (22). Anti-PRSS22 (ab197158, 1:1,000, Abcam), anti-ANXA1 (ab214486, 1:1,000, Abcam), anti-PD-1 (ab237728, 1:1,000, Abcam), β-actin (1:10,000, AC028, Company ABclonal, Inc.), and Anti-Rabbit IgG (1:5,000, GB23303, Servicebio) were used in this study. Protein was measured using the BeyoECL Plus kit (P0018S) and analyzed using an Image Lab 3.0 (BioRad Laboratories, Inc.). Anti-PRSS22 (ab197158, 1:100, Abcam) and anti-ANXA1 (ab214486, 1:100, Abcam) were used for immunofluorescence analyses.

### Statistical analysis

*P* < 0.05 was considered significant and evaluated using Student’s t-test or one-way analysis of variance (ANOVA), followed by Tukey’s post-test. Data were expressed as mean ± standard deviation (SD).

## Results

### PRSS22 expression was upregulated in GC and promoted tumor proliferation in GC mice model

**Fig. 1:**
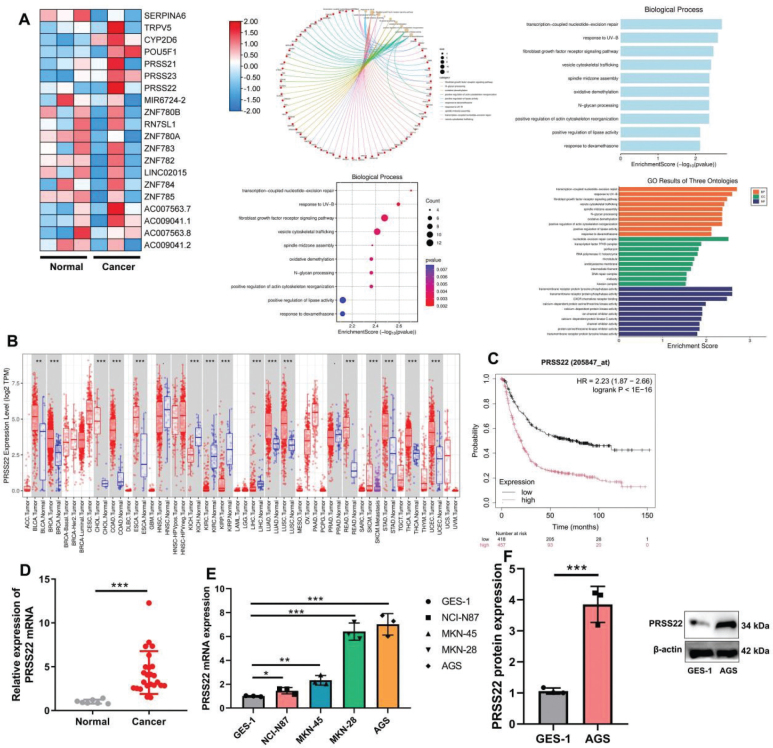
PRSS22 expression was upregulated in gastric cancer (GC). (A) The heatmap of gene expression data between normal and GC samples, and gene Ontology (GO) analysis of the differentially expressed genes (DEGs). (B) The comparison of PRSS22 expression between normal and tumor samples. (C) Overall survival (OS) curve for patients with GC with high PRSS22 expression and low PRSS22 expression. (D) The relative PRSS22 mRNA expressions in GC patients. (E) The relative PRSS22 mRNA expressions in different GC cell lines (NCI-N87, MKN-45, MKN-28, and AGS). (F) PRSS22 protein expression in AGS cell line. *P < 0.05, **P < 0.01, ***P < 0.001.

**Fig. 2:**
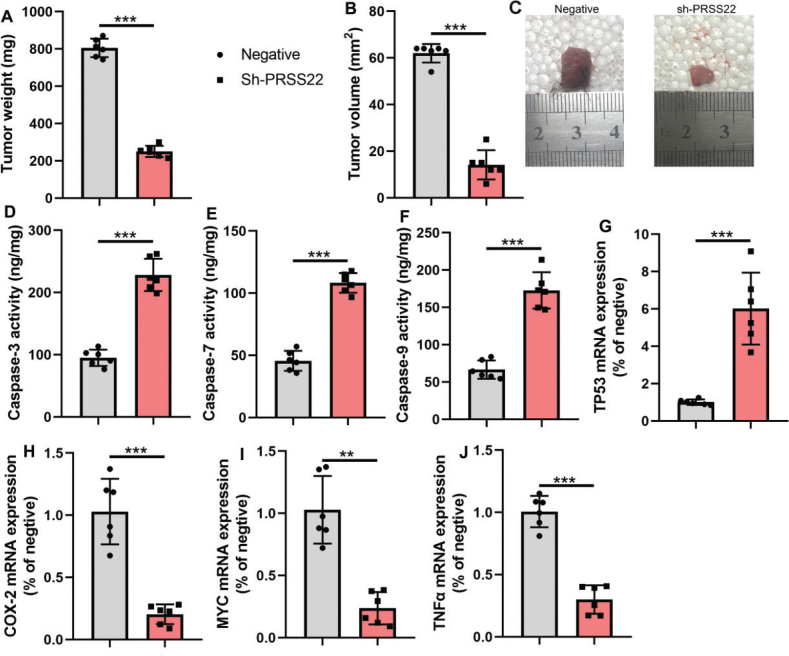
The inhibition of PRSS22 reduced tumor proliferation in mice model of gastric cancer (GC). (A, B) Tumor weight and tumor volume in GC mice treated with sh-PRSS22. (C) Tumor tissues in GC mice treated with sh-PRSS22. (D–F) Caspase-3/7/9 activity levels were determined in GC mice treated with sh-PRSS22. (G–J) qPCR analyzed the TP53/Cox2/MYC/TNF-α mRNA expressions in GC mice treated with sh-PRSS22. ^**^P < 0.01,^***^P < 0.001.

First, this study explored the disease targets for the occurrence and progression of GC using gene chip and discovered 20 DEGs, and PRSS22 expression was upregulated in patients with GC samples than normal samples (Fig. 1A). And DEGs were mainly involved in the translation-coupled nucleotide-excision repair and other biological processes. In tumors like BLCA, BRCA, ESCA, KICH, KIRC, LIHC, LUAD, and LUSC, it was found that there was significant increase of PRSS22 expressions compared with normal patients (Fig. 1B). The overall survival (OS) in patients with high PRSS22 expression was lower than that of low PRSS22 expression (Fig. 1C). Then, the relative PRSS22 mRNA expressions were upregulated in GC patients (Fig. 1D) and GC cell lines (NCI-N87, MKN-45, MKN-28, and AGS, Fig. 1E), and the PRSS22 mRNA was highest in AGS. Then, we detected the PRSS22 protein level in AGS and found that the PRSS22 protein expressions were significantly enhanced in AGS cell than GES-1 cell l line (Fig. 1F). In a GC mouse model, PRSS22 downregulation decreased tumor volume and weight, elevated the activity levels of caspase-3/7/9, reduced the mRNA expressions of Cox2, TNF-α, and Myc, and increased the TP53 mRNA expression in tumor tissues (Fig. 2).

### PRSS22 was manifested in T cells from GC patients and promoted cell growth in coculture with GC cell and T cells

**Fig. 3:**
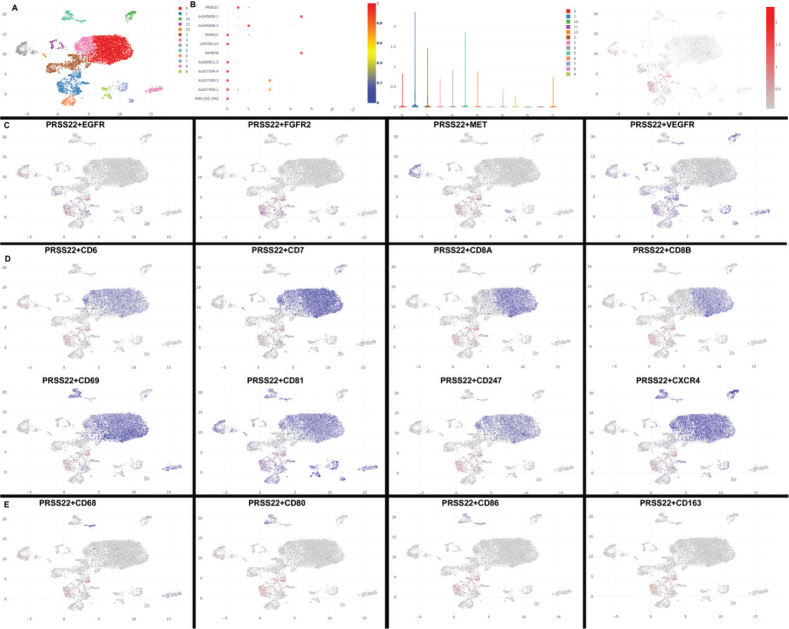
PRSS22 expressions were analyzed in T cells in patients with gastric cancer (GC). (A and B) Single-cell sequencing data for PRSS22 expressions. (C) PRSS22 expressions in tumor cells (EGFR/FGFR2/MET/VEGFR) of GC patients. (D) PRSS22 expressions in T cells (CD6/CD7/CD8A/CD8B/CD69/CD81/CD247/CXCR4) of GC patients. (E) PRSS22 expressions in macrophages (CD68/CD80/CD86/CD163, E) of GC patients.

**Fig. 4:**
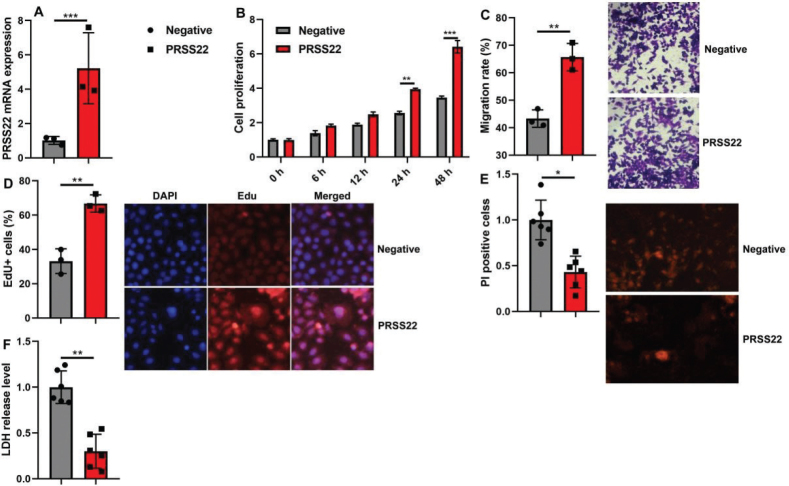
The biological functions were detected after overexpressing PRSS22 in coculture with gastric cancer (GC) cell line AGS and T cell line Jurkat cell. (A) The relative PRSS22 mRNA expression. (B) CCK-8 analyzed the cell growth. (C) Transwell determined the cellular migration. (D) EdU positivity (Scale bar: 100 μm). (E) PI positive (Scale bar: 50 μm). (F) LDH activity levels. ^*^P < 0.05, ^**^P < 0.01, ^***^P < 0.001.

**Fig. 5:**
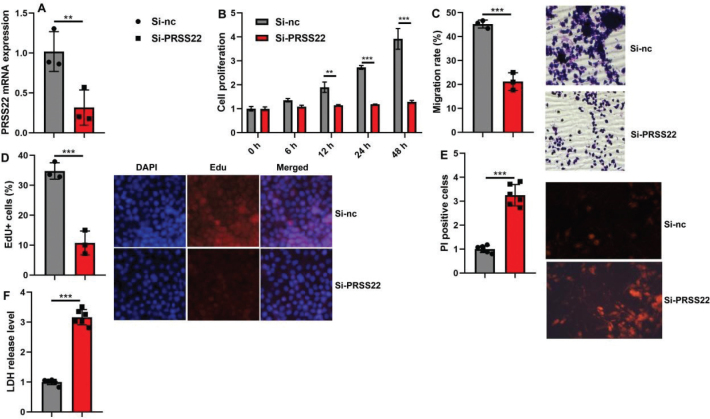
The biological functions were detected after suppressing PRSS22 in coculture with gastric cancer (GC) cell line AGS and T cell line Jurkat cell. (A) The relative PRSS22 mRNA expression in coculture. (B) CCK-8 analyzed the cell growth in coculture. (C) Transwell determined the cellular migration. (D) EdU positivity (Scale bar: 100 μm). (E) PI positive (Scale bar: 50 μm). (F) LDH activity levels in coculture. ^*^P < 0.05, ^**^P < 0.01, ^***^P < 0.001.

Then, this study delved into the mechanism of PRSS22 in GC through Single Cell Analysis. Twelve cell clusters were revealed, and PRSS22 was found to be overexpressed in cluster 1 (Fig. 3A–3B). Else, PRSS22 was found to be not expressed in cancer cells (EGFR/FGFR2/MET/VEGFR) of GC patients (Fig. 3C). PRSS22 was manifested in T cells (CD6/CD7/CD8A/CD8B/CD69/CD81/CD247/CXCR4) in GC patients (Fig. 3D). However, PRSS22 was not manifested macrophage (CD68/CD80/CD86/CD163) in GC patients (Fig. 3E). In coculture with AGS and Jurkat cell, the overexpression of PRSS22 evidently increased the relative PRSS22 mRNA expression, promoted cell proliferation, migration, and EdU positivity (Fig. 4A–D) but significantly reduced LDH activity and PI levels (Fig. 4E–F). Else, the si-PRSS22 treatment in coculture with AGS and Jurkat cell could significantly decrease the relative PRSS22 mRNA expression, suppressed cell proliferation, migration, and Edu positivity (Fig. 5A–D) but significantly promoted LDH activity and PI levels (Fig. 5E–F).

### The inhibition of PRSS22 reduced the immune evasion in GC mice model

**Fig. 6:**
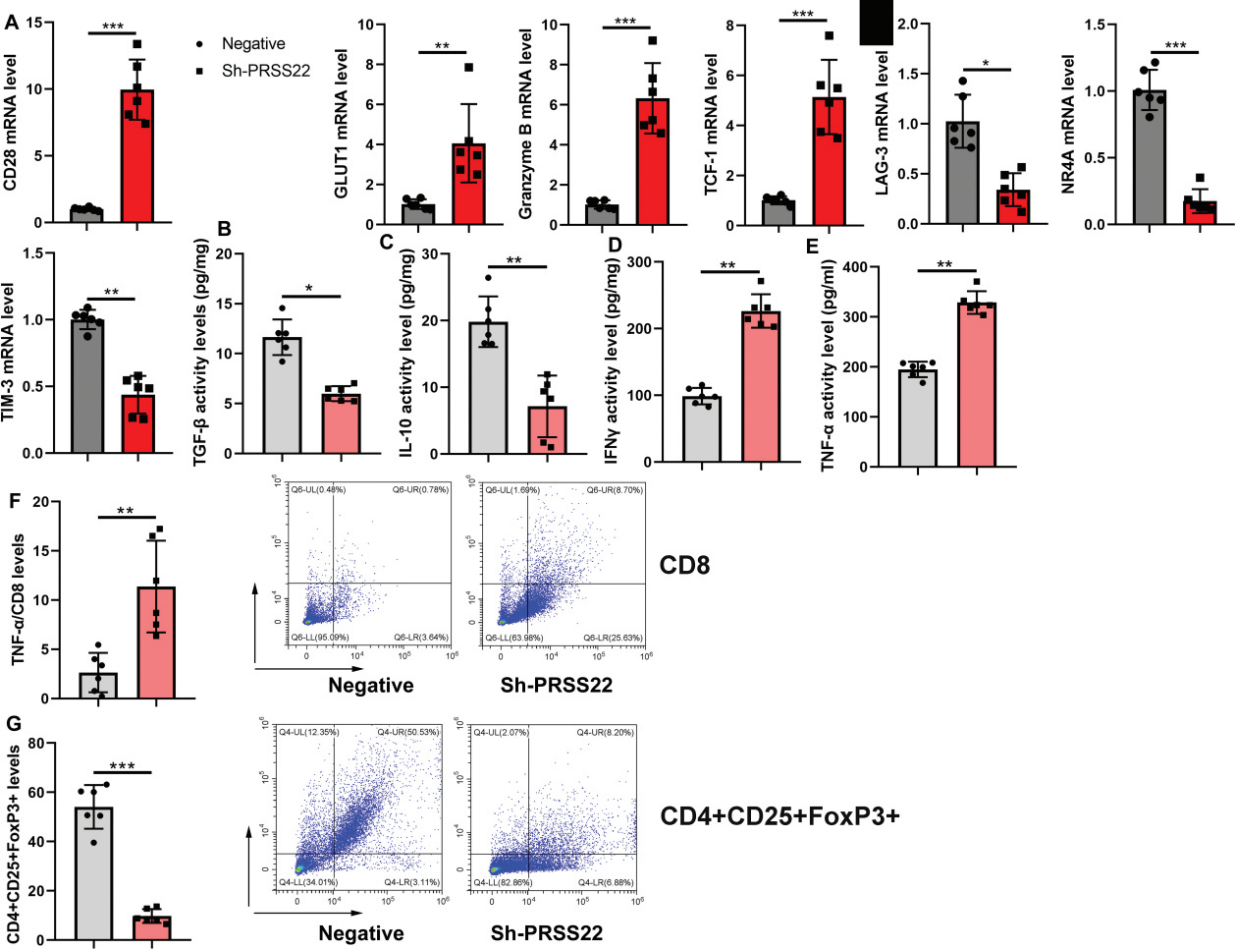
The inhibition of PRSS22 reduced the immune evasion in mice model of gastric cancer. (A) The relative CD28, GLUT1, Granzyme B, TCF-1, LAG-3, NR4A, and TIM-3 mRNA expressions. (B–E) TGF-β, TNF-α, IFNγ, and IL-10 activity levels. (F) TNFα/CD8 levels. (G) CD4^+^CD25^+^FoxP3^+^ levels. ^*^P < 0.05, ^**^P < 0.01, ^***^P < 0.001.

The effects of sh-PRSS22 on the immune evasion in the GC mice model were subsequently explored, and we observed that the sh-PRSS22 treatment could induce the relative CD28, GLUT1, Granzyme B, and TCF-1 mRNA expressions but decrease the relative LAG-3, NR4A, and TIM-3 mRNA expressions in T cells of the GC mice model (Fig. 6A). And the sh-PRSS22 treatment could decrease TGF-β and TNF-α activity levels and induce IFNγ and IL-10 activity levels in T cells of the GC mice model (Fig. 6B–E). Flow cytometer showed that sh-PRSS22 would increase TNFα/CD8 levels and reduce CD4^+^CD25^+^FoxP3^+^ levels in T cells of the GC mice model (Fig. 6F–G).

### PRSS22 promoted the immune evasion of T cells

**Fig. 7:**
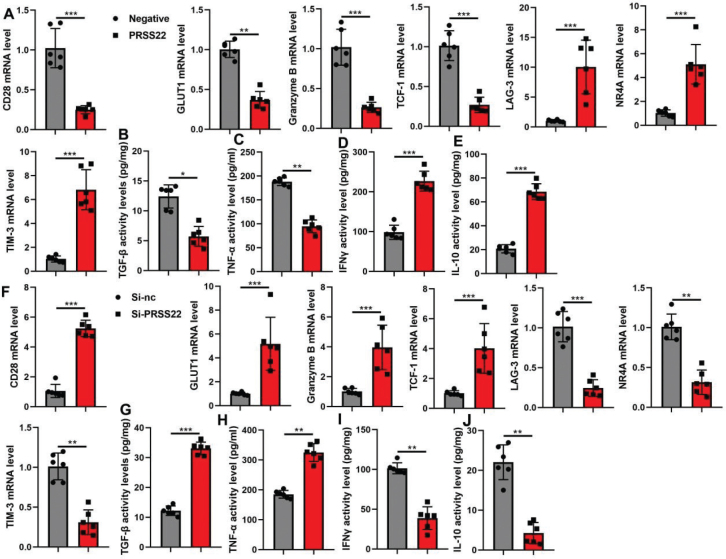
PRSS22 promoted the immune evasion of T cell. (A) The relative CD28, GLUT1, Granzyme B, TCF-1, LAG-3, NR4A, and TIM-3 mRNA expressions in the PRSS22-upregulated T cells. (B–E) TGF-β, TNF-α, IFNγ, and IL-10 activity levels in the PRSS22-upregulated T cells. (F) The relative CD28, GLUT1, Granzyme B, TCF-1, LAG-3, NR4A, and TIM-3 mRNA expressions in the PRSS22-downregulated Jurkat cell. (G–J) TGF-β, TNF-α, IFNγ, and IL-10 activity levels in the PRSS22-downregulated T cells. ^**^P < 0.01, ^***^P < 0.001.

In T cells, the PRSS22 upregulation significantly suppressed the relative CD28, GLUT1, Granzyme B, and TCF-1 mRNA expressions and evidently increased the relative LAG-3, NR4A, and TIM-3 mRNA expressions in T cells (Fig. 7A). And the PRSS22 upregulation would reduce TGF-β and TNF-α activity levels and increase IFNγ and IL-10 activity levels in T cells (Fig. 7B–E). However, the PRSS22 downregulation treatment in T cells would play a converse effect (Fig. 7F–J).

### PRSS22 suppressed ANXA1/PD-1 Signaling Pathway in T cells from GC

**Fig. 8:**
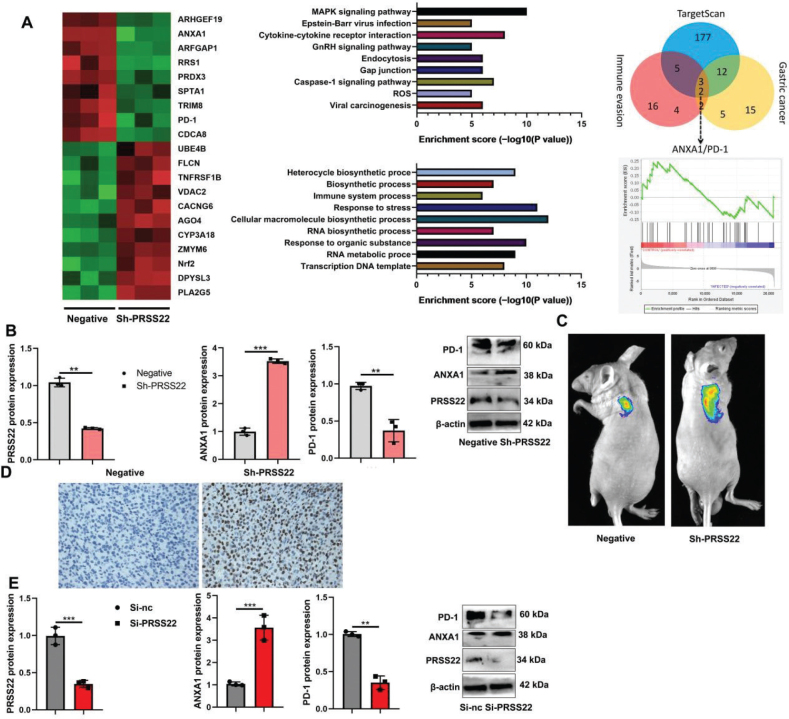
PRSS22 suppressed ANXA1/PD-1 Signaling Pathway in T cells and gastric cancer (GC). (A) The heatmap of gene expression data between the negative Jurkat cell and the sh-PRSS22 Jurkat cell, and gene ontology (GO), Kyoto Encyclopedia of Genes and Genomes (KEGG) enrichment analysis of the differentially expressed genes (DEGs), and Venn diagram of the mutual targets between immune invasion and gastric cancer. (B) The PRSS22/ANXA1/PD-1 protein expressions in tumor tissues of GC mice model with sh-PRSS22 treatment. (C) ANXA1 expression in vivo imaging. (D) Immunohistochemistry analyzed the ANXA1 expression in the GC mice model by sh-PRSS22 treatment (Scale bar: 50 μm). (E) The PRSS22/ANXA1/PD-1 protein expressions in vitro model by downregulating PRSS22. ^**^P < 0.01, ^***^P < 0.001.

**Fig. 9:**
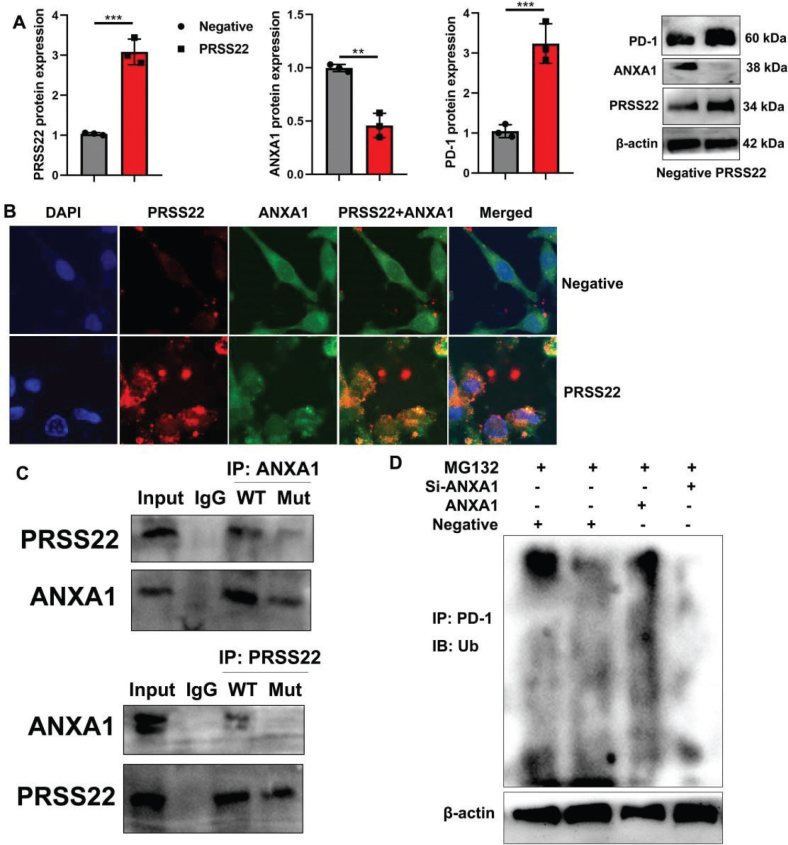
PRSS22 suppressed ANXA1-mediated degradation of PD-1 in T cells. (A) The PRSS22/ANXA1/PD-1 protein expressions in negative oe-PRSS22 T cells. (B) Immunofluorescence microscopy analyzed the PRSS22/ANXA1 expressions in negative, oe-PRSS22 T cells (Scale bar: 20 μm). (C and D) IP assay for PRSS22 protein interlinking with ANXA1 protein and PD-1 ubiquitination. ^**^P < 0.01, ^***^P < 0.001.

Then, we explored the possible mechanism of PRSS22 on immune evasion in the GC model using gene chip. The heatmap indicated the ARHGEF19, ANXA1, ARFGAP1, RRS1, PRDX3, SPTA1, TRIM8, PD-1, CDCA8, UBE4B, FLCN, TNFRSF1B, VDAC2, CACNG6, AGO4, CYP3A18, ZMYM6, Nrf2, DPYSL3, and PLA2G5 were DEGs (Fig. 8A). Go analysis reported that DEGs were primarily involved in the response to stress, cellular macromolecule biosynthetic process, response to organic substance, and other biological process (Fig. 8A). KEGG analysis indicated that DEGs were enriched in MAPK signaling pathway, Caspase-1 signaling pathway, and so on (Fig. 8A). And ANXA1/PD-1 signaling pathway might be an important signaling pathway for the effects of PRSS22 on immune evasion in the GC model (Fig. 8A). It was proved that sh-PRSS22 suppressed the PRSS22 and PD-1 protein expressions and induced ANXA1 protein expression in GC tumor tissues (Fig. 8B). In vivo imaging and immunohistochemistry showed that sh-PRSS22 induced ANXA1 expression in the GC mice model (Fig. 8C–D). In coculture with GC cell and T cells, PRSS22 downregulation suppressed PRSS22 and PD-1 protein expressions and induced ANXA1 protein expression (Fig. 8E), but PRSS22 upregulation induced PRSS22 and PD-1 protein expressions and suppressed ANXA1 protein expression (Fig. 9A). Immunofluorescence showed that PRSS22 upregulation induced expressions and suppressed ANXA1 expression of T cells (Fig. 9B). IP analysis demonstrated that the PRSS22 WT protein could interact with the ANXA1 WT protein, while the PRSS22 WT protein did not interact with the ANXA1 Mut protein, and the ANXA1 Mut protein did not link with the PRSS22 WT protein (Fig. 9C). And ANXA1 upregulation could promote the ubiquitination of PD-1 protein (Fig. 9D).

### PRSS22 induced the tumor development and the immune evasion in the coculture model by downregulating ANXA1

**Fig. 10:**
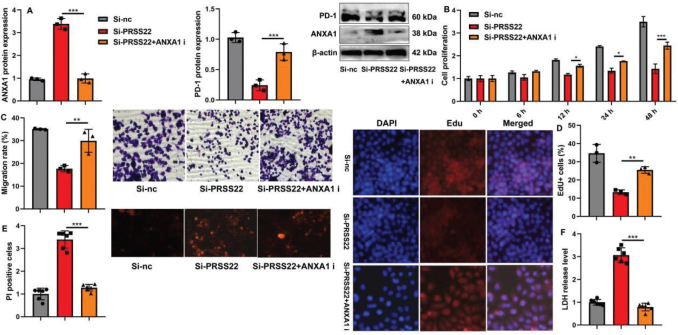
The biological functions of PRSS22/ANXA1 signaling in the coculture model with gastric cancer (GC) cell line AGS and T cell line Jurkat cell. (A) The ANXA1/PD-1 protein expressions in the coculture model with treatments of si-PRSS22 and si-PRSS22+ANXA1 i separately. (B) CCK8 analyzed the cell growth in the coculture model with treatments of si-PRSS22 and si-PRSS22+ANXA1 i separately. (C) Transwell determined the cellular migration in the coculture model with treatments of si-PRSS22 and si-PRSS22+ANXA1 i separately. (D) Edu positivity in the coculture model with treatments of si-PRSS22 and si-PRSS22+ANXA1 i separately (Scale bar: 100 μm). (E) PI positive in the coculture model with treatments of si-PRSS22 and si-PRSS22+ANXA1 i separately. (F) LDH activity levels in the coculture model with treatments of si-PRSS22 and si-PRSS22+ANXA1 i separately. ^**^P < 0.01, ^***^P < 0.001.

**Fig. 11:**
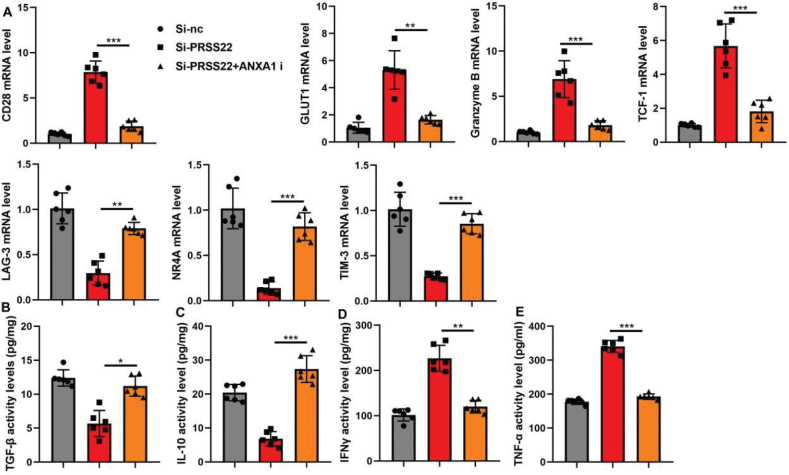
The effect of PRSS22/ANXA1 signaling on the immune evasion in the coculture model with gastric cancer (GC) cell line AGS and T cell line Jurkat cell. (A) The relative CD28, GLUT1, Granzyme B, TCF-1, LAG-3, NR4A, and TIM-3 mRNA expressions in the coculture model with treatments of si-PRSS22 and si-PRSS22+ANXA1 i separately. (B–E) ELISA analyzed the TGF-β, TNF-α, IFNγ, and IL-10 activity levels in the coculture model with treatments of si-PRSS22 and si-PRSS22+ANXA1 i separately. ^*^P < 0.05, ^**^P < 0.01, ^***^P < 0.001.

Finally, we elucidated that the role of PRSS22 regulated the ANXA1 activity on cell growth of GC and the immune evasion of T cells in the coculture model. The inhibition of ANXA1 suppressed the ANXA1 protein expression and induced the PD-1 protein expression in T cells caused by si-PRSS22 (Fig. 10A). The inhibition of ANXA1 also induced the deceased cell growth, migration, and EdU positivity but reduced the lifted LDH activity and PI levels in coculture caused by si-PRSS22 (Fig. 10B–10F). Meanwhile, the inhibition of ANXA1 suppressed the increased CD28, GLUT1, Granzyme B, and TCF-1 mRNA expressions and also suppressed the increased IFNγ and TNF-α activity levels but increased the reduced LAG-3, NR4A, and TIM-3 mRNA expressions and increased the reduced TGF-β and IL-10 activity levels in T cells caused by si-PRSS22 (Fig. 11).

## Discussion

GC is the fifth leading cause of cancer-related deaths worldwide (24). The Age-Standardized Incidence Rate by World Standard Population (ASIRW) of GC in East Asia is 32.7 per 100,000, which is higher than Eastern Europe (23.9 per 100,000) and South America (18.9 per 100,000) (25). Globally, the incidence and mortality rates of GC patients are showing a downward trend, with regional variations. In some Asian countries or regions, GC remains a major public health issue, and more than 60% of GC cases occur in East Asia (26), including China (27, 28). The existed therapies of GC patients still have some critical limitations, and there is an urgent need to find the novel target and develop new treatment methods to improve patients’ quality of life. In this study, PRSS22 mRNA and protein expressions were upregulated in GC patients or GC cells lines. The inhibition of PRSS22 reduced tumor proliferation in the mice model of GC. Song et al. showed that PRSS22 promoted the breast cancer metastasis (19). So, PRSS22 might participate in the disease progression of GC.

The tumor microenvironment (TME) is a crucial factor determining the response to immunotherapy in tumor (29). The TME of GC consists of various components such as tumor cells, immune cells [regulatory T cells (Treg), myeloid-derived suppressor cells (MDSC), and tumor-associated macrophages (TAM)], cytokines, metabolites, and exosomes (30, 31). Through dynamic interactions among these complex components, the TME exhibits both high immune sensitivity and strong immune escape ability (32). We noticed the significant PRSS22 expression of T cells in patients with GC. PRSS22 promoted cell growth of GC and the immune evasion of T cells in coculture with GC cell and T cell. Xu et al. identified that the overexpressed PRSS22 could regulate the metastasis and immune dysregulation (18). So, PRSS22 might take part in the immune evasion in GC. Future work should explore the influence of PRSS22 on other TME components, such as TAMs, MDSCs, or cytokine secretion, hereby strengthening this result.

ANXA, also known as lipocortin or lipocortin 1, can specifically bind to phosphatidylserine and participate in a series of Ca²^+^-dependent membrane-related processes, such as membrane fusion in exocytosis, vesicle transport, and Ca²^+^ channel formation (33). Its dysregulated expression is closely related to the formation and development of malignant tumors, chemotherapy resistance, etc. (34). The expression of ANXA1 is downregulated during cervical carcinogenesis, and its expression varies in different clinical stages, degrees of tissue differentiation, and lymph node status of cervical cancer. The reduced ANXA1 results in the poor prognosis of cervical squamous cell carcinoma (35). In this study, it was showed that PRSS22 reduced the ANXA1 expression in the GC tumor model and the in vitro coculture model, and the inhibition of ANXA1 recovered the effects of si-PRSS22 on cell proliferation, migration, Edu positivity, LDH activity, and PI levels of GC and the immune evasion of T cells in the coculture model with GC cell and T cell. Song et al. showed that PRSS22 promoted breast cancer metastasis by cleaving ANXA1 (19). Therefore, PRSS22 promoted immune evasion of GC by ANXA1.

PD-L1 is a typical immune surface protein that is widely expressed in different tissues of the normal body (36). The binding of PD-L1 to programmed death 1 (PD-1) on T cells triggers the programmed death of T cells, which is beneficial to maintaining the homeostasis of the immune system and is strictly regulated (37, 38). However, tumor cells can express the abnormally high levels of PD-L1, thereby inducing immune suppression. In recent years, the anti-PD-1/PD-L1 therapy has become a new method for tumor treatment by restoring the immune surveillance and antitumor functions of T cells (39). Yet, many patients still cannot receive effective outcome, with problems such as low therapeutic responsiveness, high toxicity, and severe toxic and side effects (40). Therefore, it is necessary to conduct further research on the PD-1/PD-L1 pathway (41). The exosomal PD-L1 can inhibit antitumor immunity and may be resistant to anti-PD-L1 antibody blocking, so it can be used as a potential biomarker for evaluating drug efficacy and early prediction of malignant tumors (42). We found that PRSS22 suppressed ANXA1/PD-1 Signaling Pathway in T cells through ANXA1-mediated degradation of PD-1 in T cells. Yu et al. revealed that ANXA1-derived Peptide Targeting PD-L1 Degradation reduced Tumor Immune Evasion in Various Cancers (43).

**Fig. 12:**
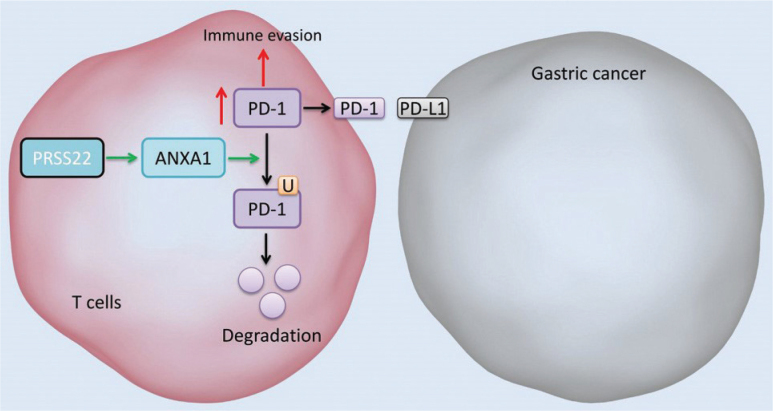
PRSS22 promotes the immune evasion of gastric cancer via inhibiting ANXA1-mediated degradation of PD-1.

In conclusion, PRSS22 suppressed ANXA1-mediated degradation of PD-1 in T cell to promote the immune evasion of GC (Fig. 12), further elucidating the role of PRSS22 in regulating the immune evasion for GC. Targeting PRSS22 is thus a potentially effective therapeutic strategy for GC.

## Data Availability

The datasets used and/or analyzed during the current study are available from the corresponding author upon reasonable request.
